# Genome Sequences of Three Novel *Bacillus cereus* Bacteriophages

**DOI:** 10.1128/genomeA.01118-13

**Published:** 2014-01-23

**Authors:** Julianne H. Grose, Jordan D. Jensen, Bryan D. Merrill, Joshua N. B. Fisher, Sandra H. Burnett, Donald P. Breakwell

**Affiliations:** Department of Microbiology and Molecular Biology, Brigham Young University, Provo, Utah, USA

## Abstract

The *Bacillus cereus* group is an assemblage of highly related firmicute bacteria that cause a variety of diseases in animals, including insects and humans. We announce three high-quality, complete genome sequences of bacteriophages we isolated from soil samples taken at the bases of fruit trees in Utah County, Utah. While two of the phages (Shanette and JL) are highly related myoviruses, the bacteriophage Basilisk is a siphovirus.

## GENOME ANNOUNCEMENT

*Bacillus cereus* group bacteria include the eponymous species as well as the closely related *B. anthracis*, *B. thuringiensis*, *B. mycoides*, *B. pseudomycoides*, and *B. weihenstephanesis*. These firmicute bacteria are commonly found in soil, but they also cause a variety of human infectious diseases. For example, when found in food, *B. cereus* is frequently associated with emetic and diarrheal forms of gastroenteritis (for recent reviews, see references 1 and 2). In soil, *B. thuringiensis* infects the insect pests of many crops (for recent reviews, see references 3 through 5). The genomes of approximately 42 *B. cereus* group bacteriophage and prophage genomes can be found on GenBank. While the isolation of bacteriophages may aid in the treatment of food products, understanding the structures of their genomes may further define not only the evolution of the bacteriophages themselves, but also that of their *Bacillus cereus* group hosts, since many of the phages have shown significant cross-infectivity (6–8). Of the 42 *B. cereus* group bacteriophages, 24 were isolated using *B. cereus* as the host. Here we announce the genome sequences of three novel *B. cereus* bacteriophages, Basilisk, Shanette, and JL.

The three phages were isolated using enrichment cultures of soil samples collected in and around Provo, UT, using a locally isolated host strain (*B. cereus* BC7003). Following at least three plaque purifications, a high-titer phage lysate was prepared and genomic DNA was extracted. Briefly, high-titer lysates were incubated with 5  µg/ml RNase and 10 µg/ml DNase for 30 min at 37° C and treated with 100 µg/μl proteinase K at 52°C for 1 h. Sequencing was accomplished using 454 pyrosequencing (Roche). Multiple contigs were formed using Newbler version 2.6 (Roche Diagnostics, Branford, CT) and CONSED version 19 (9). Assembly was completed and checked using Gepard 1.30 (10). Although the physical ends and the packing and replication strategies of the phage DNA were not determined, the manual finishing and overlapping contigs assembled the genome sequence into an apparently circular genome. Base one was selected using the noncoding region upstream of the terminase gene. The putative open reading frames (ORFs) of each genome were predicted and annotated using DNA Master (http://cobamide2.bio.pitt.edu). The criteria used to assign potential ORFs were GeneMark HHM and Glimmer autoannotation, BLAST alignment *E* values of <0.001, coding potential from GeneMark (11) using *B. cereus* ATCC 14579, start codon sequences, and Shine-Dalgarno (SD) scores of >200 using the Karlin position-specific scoring matrix (PSSM) for moderately to highly expressed genes. Additionally, tRNAs were predicted using the ARAGORN (12) program. From transmission electron microscopy analysis, it was determined that Shanette and JL were of the family *Myoviridae* and Basilisk of the family *Siphoviridae*.

### Nucleotide sequence accession numbers.

The GenBank accession numbers, sequencing fold coverages, genome sizes, numbers of putative ORFs, numbers of tRNA genes, and GC content percentages for these bacteriophages are summarized in Table 1.

**TABLE 1:** Data for three novel *B. cereus* bacteriophage genomes

Phage name	GenBank accession no.	Sequencing fold coverage	Length (bp)	No. of ORFs	No. of tRNAs	GC content (%)
Basilisk	KC595511	74.42	81,790	140	2	33.9
Shanette	KC595513	230.81	138,877	223	3	40.8
JL	KC595512	56.26	137,918	222	4	40.8
